# Extensor hood injuries in elite boxers: injury characteristics, surgical technique and outcomes

**DOI:** 10.1177/17531934221123139

**Published:** 2022-09-15

**Authors:** Gulraj S. Matharu, Ian T. Gatt, Rachel Delaney, Michael Loosemore, Michael J. Hayton

**Affiliations:** 1 Musculoskeletal Research Unit, Bristol Medical School, University of Bristol, Southmead Hospital, Westbury-on-Trym, Bristol, UK; 2 Physiotherapy Department, English Institute of Sport, Sheffield, UK; 3 Hand Therapy Department, The OrthTeam Centre, Manchester, UK; 4 Institute of Sport, Exercise and Health (ISEH), University College London, London, UK; 5 The Upper Limb Unit, Wrightington Hospital, Lancashire, UK

**Keywords:** Extensor hood injury, elite athlete, surgical repair, outcomes, return to sport

## Abstract

We describe our experience of managing extensor hood injuries in boxers (57 fingers). The diagnosis was mostly clinical, with imaging only if the diagnosis was equivocal. The middle (61%) and index (26%) digits were most frequently injured. On exploration, 26% had no hood tear, however all required tenolysis from the adherent capsule. Of 42 hood tears, 15 were central splits between adjacent extensor tendons in the index or little fingers,15 tears were on the ulna side of the extensor tendon and 12 tears were on the radial side. A pseudobursa was encountered in 35%, capsular tears in 28% and chondral injury in one patient. Longitudinal curved metacarpophalangeal joint incisions were used, with hood repair performed in flexion using a locked running suture. Mean postoperative metacarpophalangeal joint flexion was 90°. Ninety-eight per cent returned to the same level of boxing at a mean of 8 months (range 1–24) from surgery. One finger was revised for re-rupture 6 months later. A reproducible technique for treating these injuries is described, with patients able to return to boxing with little risk of complications.

**Level of evidence:** IV

## Introduction

Hand injuries are the commonest type of injury sustained by amateur and professional boxers and lead to the most time lost from training and competition (Loosemore et al., 2015). The high prevalence of hand injuries in boxers represents a true sport-specific epidemic (Melone et al., 2009). However this increased risk of hand injury is not surprising given the high forces transmitted through a clenched fist (Smith et al., 2000).

Extensor hood injuries represent 16% of all hand and wrist injuries in boxers. These can include a tear of the extensor hood, damage to the joint capsule, synovitis of the joint or any combination of these (Loosemore et al., 2017). These injuries in professional and amateur boxers can lead to significant problems with sporting hand function, with extensor hood tears leading to an average of 110 days lost from sport due to injury (Loosemore et al., 2017). Patients with these injuries have pain on impact and are unable to compete, and therefore commonly seek medical attention at this stage. Although surgery is often needed to manage extensor hood tears in boxers, little is known about the surgical management and subsequent outcomes in these high-demand athletes, including the ability to return to their sport postoperatively. Most reports are from small series, which include fewer than 15 patients and with short follow-up periods (Arai et al., 2002; Bents et al., 2003; Hame and Melone, 2000; Loosemore et al., 2009; Melone et al., 2009; Posner and Ambrose, 1989).

We describe a single-surgeon’s (MJH) experience of managing extensor hood injuries in elite (professional or international amateur level) boxers, with specific focus on the injury characteristics, surgical technique and outcomes.

## Methods

This prospectively designed cohort study included consecutive elite boxers undergoing surgery for clinically suspected extensor hood injuries between 2008 and 2021. The inclusion criteria were boxers with painful metacarpophalangeal joints (MCPJs) and extensor hood tenderness, which had affected their ability to box and compete. The exclusion criteria were patients presenting with other diagnoses, such as fractures, sprains and carpometacarpal instability. Referrals were either from the medical team of the relevant amateur national sporting organization or, in the case of professionals, from the boxer themselves. All operation were done by an experienced sports hand surgeon (MJH), with all data prospectively collected and recorded in an electronic patient database (Loosemore et al., 2009). Ethical approval was not sought for the study because patients were assessed and followed-up in-line with the surgeon’s routine clinical care with no deviations from normal clinical practice. This study was completed in accordance with the Helsinki Declaration as revised in 2013.

### Data collected

Data were prospectively collected on demographics (age, sex, hand dominance, boxing stance), previous treatment (steroid injections or surgery, or both), imaging, and injury characteristics (hand, digit(s) injured, presence of a pseudobursa, extensor hood tear including the position of the tear within the hood (radial, ulna, central), capsular tear, and chondral damage). Data were also prospectively collected on postoperative outcomes including MCPJ flexion, return to boxing (and whether or not this was at the previous level of competition), any complications and further surgery. Return to boxing was assessed from a number of sources, including the patients at their follow-up appointments, through the medical staff and from online records of professional boxing events through BoxRec (https://boxrec.com/en). BoxRec is a dedicated website that accurately holds the current and historical fighting records of professional and amateur boxers, and currently holds information on over 2.3 million fights.

### Diagnostic criteria

The clinical presentation of extensor hood injuries is usually that of pain over the affected MCPJ after either a single forceful punch against an opponent, or a gradual onset of pain after repeated impact while boxing. Swelling and bruising may be present acutely. There is often pain on palpation over either side of the extensor hood, with the knuckle feeling boggy. Palpable crepitation can sometimes be felt over the area and is likely to indicate a pseudobursa (discussed below). There may also be reduced flexion. Extensor tendon instability is occasionally present, seen when the tendon subluxes during flexion of the MCPJ.

The diagnosis was mostly clinical, with imaging (dynamic ultrasound or magnetic resonance imaging (MRI)) reserved for equivocal cases. In many cases in which imaging was available, it had been done before referral for a surgical opinion and did not add useful clinical information when there was a consistent history and examination. All patients were advised before the operation of the possibility no tear might be found, and that they would have a surgical wound and a short period of immobilization afterwards if this was the case.

### Surgical technique

**Figure 1.**
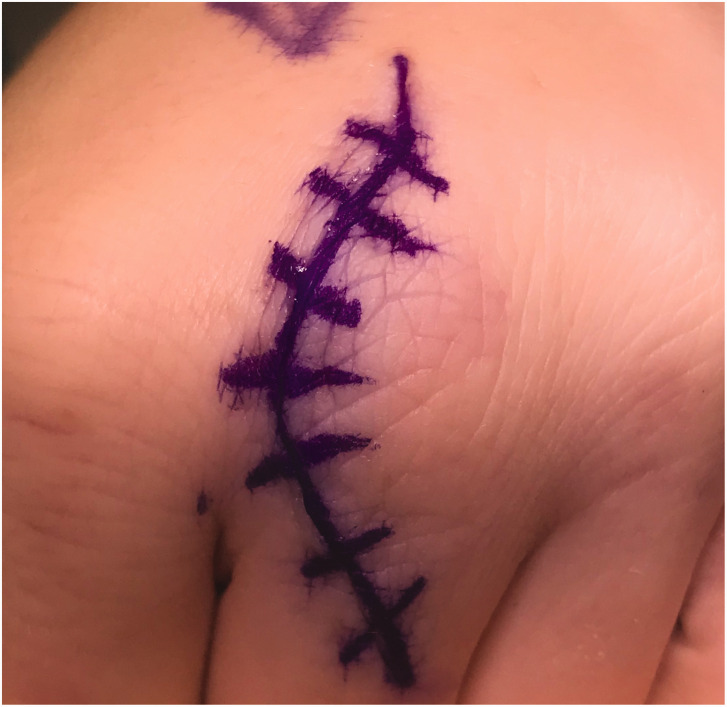
Skin incision. A curved longitudinal incision avoids a scar over the striking point of the knuckle.

**Figure 2.**
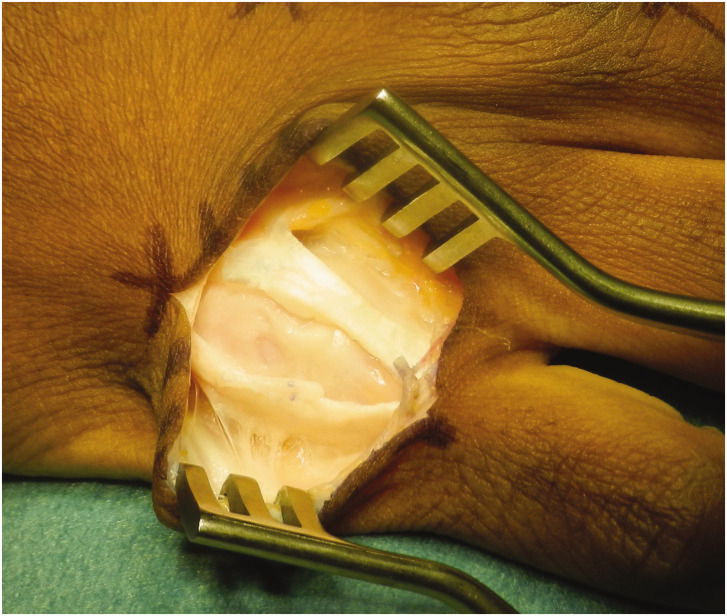
Little finger extensor tear. Longitudinal split in the extensors of the little finger between extensor digitorum and extensor digiti minimi tendons.

**Figure 3.**
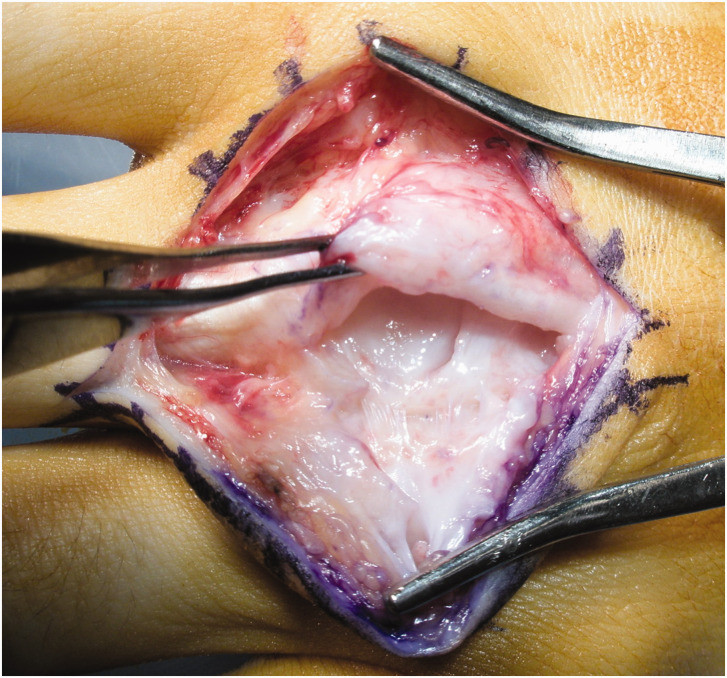
Chronic tear. Chronic extensor hood tears, even with rolled up edges, can often be debrided and a primary suture repair achieved.

Most operations are now done wide-awake under local anaesthesia with no tourniquet (WALANT). Longitudinal curved incisions over the ulnar side of the MCPJs are used to avoid scars directly over the metacarpal head (Figure 1). If two adjacent digits require exploration, a curved “
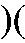
“ incision is made. The skin flap is carefully elevated off the extensor hood and inspected for any pseudobursa and longitudinal splits in the extensor hood on either side of the tendon (Figures 2 and 3). If a hood tear is found, the tear in the hood is extended longitudinally.

**Figure 4.**
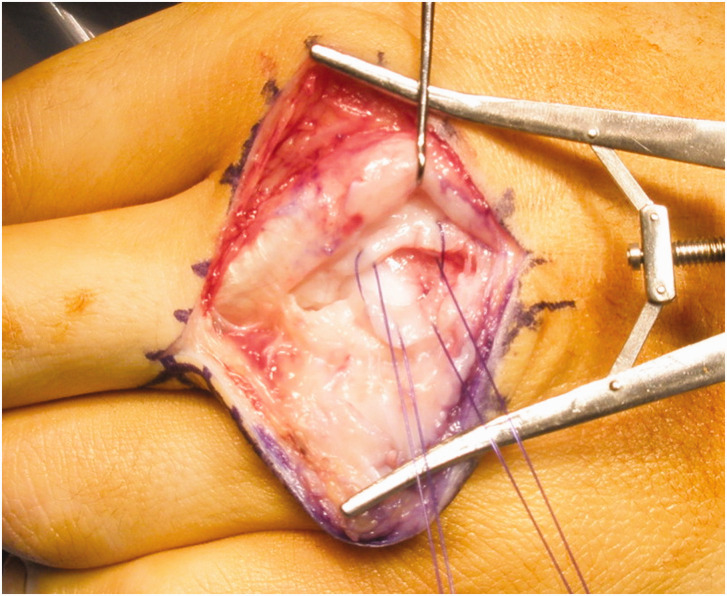
Capsule repair. The capsule should only be repaired with the metacarpophalangeal joint in 90° of flexion. If this cannot be achieved, the capsule should be left open to heal by secondary intention.

**Figure 5.**
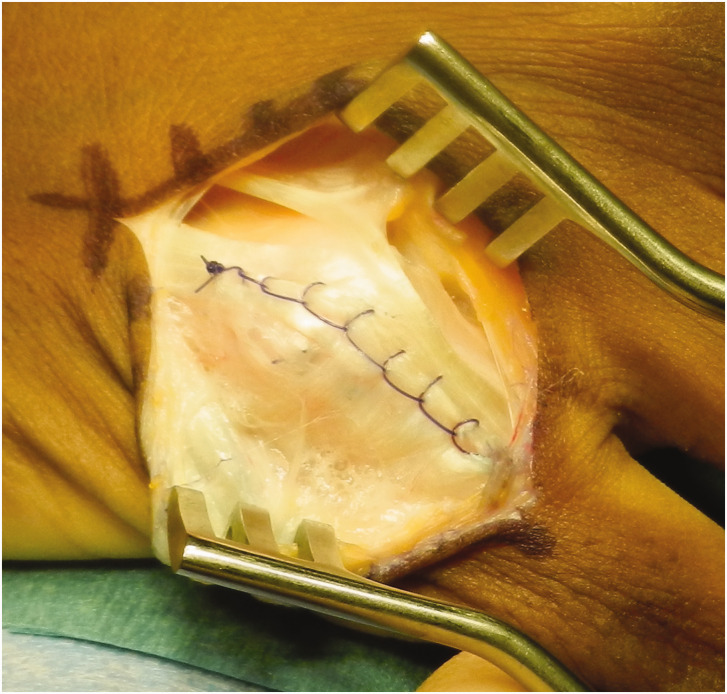
Repair of little finger extensor tear. Little finger extensor tear (from Figure 2) repaired with a running locked suture inserted with the metacarpophalangeal joint at 90°.

The capsule is then exposed and assessed for a tear. A torn capsule is repaired only if this can be done with minimal tension in 90° of MCPJ flexion (Figure 4). If not, such as in cases with large capsular defects, the capsule can be left open. The extensor hood is repaired in full flexion using a locked polydioxanone (PDS) running suture (3–0 or 4–0 depending on patient size) with the knot buried on the deep surface of the tendon and above the capsule (Figure 5).

### Rehabilitation

Rehabilitation is supervised by a hand therapist or physiotherapist. The patient is discharged in a bulky bandage and is advised to carry out early gentle finger movements inside the bandage. Gentle active flexion and extension of all finger joints is started within the first week although patients are advised not to passively force the MCPJs into flexion and to avoid active power gripping. During the first 2 weeks the patient is advised to actively move the MCPJs with the interphalangeal joints in full extension, and to practise MCPJ extension with the interphalangeal joints in flexion, and also flexion of the interphalangeal joints with the MCPJs in neutral.

**Figure 6.**
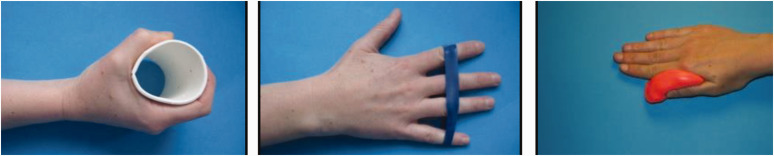
Strengthening exercises. After 4 weeks the patient may start isometric contractions of the long flexor tendons and strengthening the intrinsic muscles.

After 4 weeks the patient may start isometric contractions of the long flexor tendons, and grip strengthening for the intrinsic muscles (Figure 6). Progressive active hand and wrist loading continues between 4 and 8 weeks as the repair heals. Between 8 and 12 weeks the boxer can gradually resume light impact through the repair. Progressing loading in a systematic manner starts with water punching bags, progressing to normal bags (from softer to harder), then pads, technical sparring and eventually open sparring can begin if there are no adverse reactions at each stage. In our experience the period from initial impact to full impact in sparring is around 4–6 weeks.

## Results

### Patient and injury characteristics

Fifty-seven fingers underwent surgery (45 patients). When more than one finger was operated on they were treated at the same surgical procedure. The mean age was 24 years (range 16–35) and 95% (*n* = 54) were male; 95% (*n* = 54) were right-hand dominant and fought with an orthodox stance (leading with left hand), the rest being southpaws (leading with right hand). Symptom duration (i.e. from initial injury to the time of surgery) varied with a mean of 8.6 months (range 0.5–30).

Injuries most commonly involved the right hand (65%, *n* = 37). In the 54 orthodox fighters, 67% (*n* = 36) had right-hand injuries. In the three southpaws, two had left-hand injuries. The most frequently injured digits were the striking digits: middle (61%; *n* = 35), index (26%; *n* = 15), little (11%; *n* = 6) and ring (2%; *n* = 1).

Preoperative imaging was obtained in 39% (*n* = 22). The most common modality was MRI alone in 11, followed by ultrasound alone in nine and both ultrasound and MRI in two. In two cases imaging showed an extensor hood tear but no tear was found on surgical exploration; in three cases imaging was normal but extensor hood tears were identified intraoperatively. Between one and four preoperative steroid injections into the MCPJ (a total of 18 injections in the cohort) had been given in 23% (*n* = 13) of the digits. Most injections (16/18) had been administered by other practitioners before referral to our team and given between 6 weeks and 6 months before surgery. Most patients had symptomatic improvement for a short period, but symptoms recurred soon after starting full impact training.

On exploration, 26% (*n* = 15) had no hood tear but all required tenolysis from the adherent underlying capsule. In all cases there was some degree of adhesion formation between either the tendon and the skin or the tendon and the capsule. In the patients without a hood tear, three had a pseudobursa, and none had a capsular tear or chondral injury.

Of 42 hood tears, 15/42 were central splits between adjacent extensor tendons in the index or little fingers (i.e. between extensor indicis (EI) and extesnor digitorum (ED), or between extensor digiti minimi (EDM) and ED respectively), 15/42 were on the ulnar side, and 12/42 were radial. A pseudobursa was encountered in 35% (*n* = 20) of all cases in this study, and in 17/42 patients with hood tears. Capsular tears were found in 28% (*n* = 16) of all cases in this study, and in 16/42 patients with hood tears. A chondral injury was identified in only one case in this study, in a patient who had both a hood tear and a capsular tear. In the 16 capsular tears, tension free capsular repair was possible in flexion in ten cases. Extensor hood closure could be done primarily in all cases, without the requirement to use autografts to repair any defects.

### Postoperative outcomes

The mean postoperative follow-up period was 4.1 years (range 0.1–10.9), with all but one patient completing the rehabilitation protocol. The outcomes subsequently presented therefore relate to 56 fingers undergoing surgery in 44 patients. The mean postoperative MCPJ flexion was 90° (range 75°–95°), with all the extensor tendons tracking centrally during motion. The one patient who had flexion of 75° had received four steroid injections elsewhere into the MCPJ before having surgery. Of the 44 patients, 43 patients returned to the same competition level of boxing at a mean of 8 months (range 1–24) after surgery. The patient with the chondral injury was the only one that did not return to boxing and subsequently became a boxing trainer.

One finger had revision surgery for re-rupture occurring 6 months after extensor hood repair. It was later found that the patient had received a steroid injection to the affected MCPJ 6 weeks before surgery. There were no other reoperations or complications in other patients including the 15 patients who were explored with no tears identified at operation, all of whom had postoperative MCPJ flexion of 90° and returned to the same competition level of boxing.

## Discussion

After the surgical treatment and rehabilitation protocol for extensor hood injuries that we have described and which have been developed by the lead surgeon, patients can expect to achieve a full recovery and return to boxing at the same level as previously with few complications.

The anatomy of the extensor hood is complex (Mitsuzawa et al., 2021). Extensor hood injuries in boxers represent serious injuries that historically were career ending with boxers retiring with ‘weak hands’. In all series, including ours, they occur in young men, however we have seen an increase in women as the popularity of the sport in women grows (Arai et al., 2002; Bents et al., 2003; Hame and Melone, 2000; Loosemore et al., 2009; Melone et al., 2009; Posner and Ambrose, 1989). Early surgical repair has been recommended in this high-demand group to restore extensor integrity, prevent irreparable tissue damage and maintain optimal joint function, given that degenerative joint disease may ensue without surgery (Melone et al., 2009). In our series, 88% of extensor hood injuries involved the middle and index fingers, which is not surprising given they are the longer striking digits. When affecting the other digits, the tears were split equally between being radial and ulnar in the location in the hood. This is in contrast with the only other large series of extensor hood injuries from the United States, which included 44 procedures (Melone et al., 2009), and in which most tears were radial (60%), with only 9% being central tears. Furthermore, capsular tears were more commonly seen (73%) compared with our series (32%). The differences in injury patterns may be multifactorial, such as in the way fighters are trained in different countries. Also the previous series was of patients operated on over a decade ago, whereas our series was more recent and may reflect advances in injury prevention.

We believe that the clinical assessment provides all the information necessary to make a diagnosis. We therefore do not routinely use imaging because of the associated costs and lack of additional information it provides after clinical assessment.

We pay particular attention to pseudobursa debridement, which often overlies the underlying hood tear. In cases with an intact extensor hood we now inject the underlying MCPJ to determine whether the capsule is intact or breached by assessing syringe backfill. It is particularly important to close the capsule and hood in flexion. If the capsule cannot be closed in full flexion we simply leave it alone and have noted no adverse outcomes, similar to what others have reported (Melone et al., 2009). In our series all extensor hood tears could be repaired primarily tension free in flexion.

Initially repairs were protected in a dorsal 90° back slab and then splinted for 4–6 weeks. This was largely disregarded by professional athletes with no detriment to outcome; therefore we no longer immobilize, although others do (Lin and Strauch, 2014; Melone et al., 2009). We aim to achieve early full range of motion (ROM) while the underlying repair is healing. As repairs are done in full flexion without tension using WALANT the patients can see the ROM on the table and know that it can be maintained postoperatively.

Revision surgery was done in one case. The re-tear was repaired successfully with no further problems and the patient returned to boxing at the same competition level; however, the steroid injection to the affected MCPJ 6 weeks before surgery may have contributed to early repair failure. We are now more cautious about recent steroid injections, and prefer a minimum interval of 12 weeks between injections and surgery.

The study strengths include being a large series of elite boxers with their activity and competitions accurately tracked by BoxRec, which requires passing a medical assessment from the relevant medical boxing board of controls before competing. Limitations include the possibility that the experience and results of one surgeon may not be generalizable to all surgeons undertaking these procedures, especially those with limited experience. It was not possible to have the same protocol for all patients, given that operations were carried out over 13 years and the surgeon’s protocols changed over time, such as reducing the postoperative immobilization period. Some patients had short follow-up periods, which is a common problem in young athletes, and must be considered when interpreting our results. However, all results have been presented here regardless of the follow-up time of each patient to avoid introducing bias. Finally, despite having return-to-boxing data for all patients, validated patient-reported outcome measures were not available, neither were objective data like grip and pinch strength. Although we recognize this as a limitation, it is suspected that these data would not provide substantial information in addition to the return to boxing data.
